# Impact of geographic distance on appraisal delay for active TB treatment seeking in Uganda: a network analysis of the Kawempe Community Health Cohort Study

**DOI:** 10.1186/s12889-018-5648-6

**Published:** 2018-06-26

**Authors:** Kyle Fluegge, LaShaunda L. Malone, Mary Nsereko, Brenda Okware, Christian Wejse, Hussein Kisingo, Ezekiel Mupere, W. Henry Boom, Catherine M. Stein

**Affiliations:** 1 Department of Population and Quantitative Health Sciences, Case Western Reserve University, 10900 Euclid Avenue, Cleveland, OH 44106 USA; 2 Present address: Office of Strategic Data Use, New York City Department of Health and Mental Hygiene, 42-09 28th Street, Long Island City, NY 11101-4132 USA; 3 Present address: Institute of Health and Environmental Research, Cleveland, OH 44118 USA; 4 Tuberculosis Research Unit, Case Western Reserve University, 10900 Euclid Avenue, Cleveland, OH 44106 USA; 5 Case Western Reserve University Research Collaboration, Kampala, Uganda; 6 Department of Infectious Diseases, Institute for Clinical Medicine / Center for Global Health, Department of Public Health, Aarhus University, Aarhus, Denmark; 7 Department of Pediatrics and Child Health College of Health Sciences, Makerere University, Kampala, Uganda; 8 Department of Population and Quantitative Health Sciences and Tuberculosis Research Unit, Case Western Reserve University, 10900 Euclid Avenue, Cleveland, OH 44106 USA

**Keywords:** Healthcare access, *Mycobacterium tuberculosis*, Treatment delay, Health services research

## Abstract

**Background:**

Appraisal delay is the time a patient takes to consider a symptom as not only noticeable, but a sign of illness. The study’s objective was to determine the association between appraisal delay in seeking tuberculosis (TB) treatment and geographic distance measured by network travel (driving and pedestrian) time (in minutes) and distance (Euclidean and self-reported) (in kilometers) and to identify other risk factors from selected covariates and how they modify the core association between delay and distance.

**Methods:**

This was part of a longitudinal cohort study known as the Kawempe Community Health Study based in Kampala, Uganda. The study enrolled households from April 2002 to July 2012. Multivariable interval regression with multiplicative heteroscedasticity was used to assess the impact of time and distance on delay. The delay interval outcome was defined using a comprehensive set of 28 possible self-reported symptoms. The main independent variables were network travel time (in minutes) and Euclidean distance (in kilometers). Other covariates were organized according to the Andersen utilization conceptual framework.

**Results:**

A total of 838 patients with both distance and delay data were included in the network analysis. Bivariate analyses did not reveal a significant association of any distance metric with the delay outcome. However, adjusting for patient characteristics and cavitary disease status, the multivariable model indicated that each minute of driving time to the clinic significantly (*p* = 0.02) and positively predicted 0.25 days’ delay. At the median distance value of 47 min, this represented an additional delay of about 12 (95% CI: [3, 21]) days to the mean of 40 days (95% CI: [25, 56]). Increasing Euclidean distance significantly predicted (*p* = 0.02) reduced variance in the delay outcome, thereby increasing precision of the mean delay estimate. At the median Euclidean distance of 2.8 km, the variance in the delay was reduced by more than 25%.

**Conclusion:**

Of the four geographic distance measures, network travel driving time was a better and more robust predictor of mean delay in this setting. Including network travel driving time with other risk factors may be important in identifying populations especially vulnerable to delay.

**Electronic supplementary material:**

The online version of this article (10.1186/s12889-018-5648-6) contains supplementary material, which is available to authorized users.

## Background

Tuberculosis (TB) remains a global disease burden, especially for developing countries with high prevalence of individuals co-infected with HIV. In 2015, Uganda had an overall TB incidence rate of 202 per 100,000 and 66 per 100,000 among HIV-positive individuals. This rate placed the country in the top twenty of disease burden among all countries assessed for the double epidemics of TB and HIV [1]. Long delay in starting treatment, especially among HIV-positive TB patients, has been associated with unfavorable treatment outcomes [2]. Previous research has identified a common reason for delay: many patients, including those co-infected, view initial symptoms as not serious [3, 4] and, in some cases, not even reflective of TB [5].

**Fig. 1:**
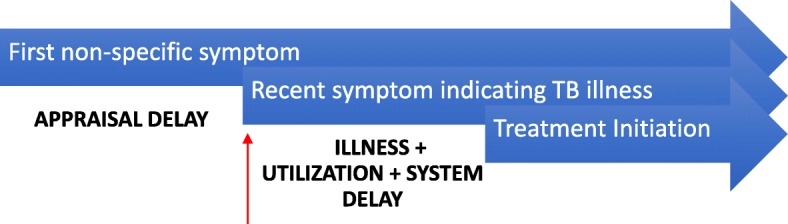
Appraisal delay is the time a person takes to evaluate a symptom as a sign of illness. Illness delay is the time the person takes from the first sign of illness until deciding to seek professional medical care. Utilization delay is the time from the decision to seek care until the consult at a health facility. System delay is the time from the first consultation to initiation of treatment. The red arrow indicates the appraisal date, at which time the patient recognizes possible TB as the explanation for his or her symptoms

Appraisal delay is the time a patient takes to consider a symptom as not only noticeable, but a sign of illness [6]. The occurrence over time of more than one symptom is an indicator to many patients of the presence of illness necessitating medical intervention. Multi-symptom appraisal delay has been suggested when considering symptom clusters in chronic disease [7]. In the case of TB, many early symptoms are non-specific and therefore not immediately perceived as signals of disease among individuals who experience them [8]. However, the co-occurrence of symptoms can influence patients’ disease perception. For example, cough is often not recognized as possible TB unless accompanied by more serious symptoms like hemoptysis and weight loss [9, 10], after which patients are more likely to seek health care [11]. Symptom duration is defined as the number of days from the first day of onset of any symptom attributed to tuberculosis until the first day of appropriate TB therapy [12]. This definition frequently encompasses illness, utilization and system delay (see Fig. 1). Several studies of TB patients have considered this definition when deriving a quantitative (generally binary) measure of patient delay [13–16]. However, such a definition obscures the occurrence of existing, albeit nonspecific, symptoms that preceded the appraisal date (see Fig. 1).

Our goal was to investigate whether distance to healthcare facility influences the patient’s appraisal delay. We did so by assessing the period before that used to typically define delay in the TB literature, what we refer to as the appraisal interval, a period when the illness is perceived by the patient to be either non-existent and/or non-threatening. Stock [17] examined the impact of distance to health facility on health care utilization in sub-Saharan Africa. He discovered the association depended upon illness perception; the more serious the disease (i.e., TB), the less distance to facility impeded utilization. However, this finding was based on data from the 1970s, a pre-HIV/AIDS era in which the TB burden was comparatively lower [18] and stigma not as great. In recent decades, however, as technology has enhanced our ability to categorize not only disease but also its severity, research findings generally flip Stock’s assessment [19]. We hypothesized that a greater distance to clinic extends the appraisal interval, contributing to a longer period of overall delay. If confirmed, it restores Stock’s [17] initial finding that a patient’s perception of illness severity is an important modifier of the relationship between distance to health facility and treatment utilization.

To measure distance, we considered Euclidean distance and network travel time. Euclidean distance, owing to its computational simplicity, has been commonly used to measure distance and is calculated as the straight-line distance between two geographic locations [20]. Network travel time, derived from network analysis, uses distance and speed to systematically create the fastest (or least costly) travel time route between two geographic locations in a given road network [20]. Deriving a more sophisticated measure of geographic distance may allow a more accurate assessment of access to health services, leading to more effective interventions.

## Methods

### Setting

The data from this study were obtained from a longitudinal cohort study called the ‘Kawempe Community Health Study’ (KCHS), which enrolled households from April 2002 to July 2012 in Kampala, Uganda [21]. Participants resided within Kawempe and contiguous divisions, representative of other sub-Saharan low-resource settings.

### Study participants

Eligible participants (index cases) were 18 years or older, had an initial pulmonary TB diagnosis that was confirmed based on growth of *Mycobacterium tuberculosis* in culture, resided in Kawempe Division or contiguous divisions for at least three consecutive months and provided HIV testing and informed consent. Referral sources included direct self-referral to the Ugandan National Tuberculosis and Leprosy Program (NTLP), community sensitization outreach programs, community/private clinics or some other source.

### TB screening

Eligible patients received a baseline evaluation consisting of a standard history, physical examination and a comprehensive clinical work-up, which included chest radiography and acid-fast bacilli (AFB) sputum smear/culture. Patients were asked questions by a trained nurse or counselor, who then recorded the patient’s responses onto the case report forms. Patients were instructed to return to the clinic in 7 days to determine enrollment into the study. Enrolled patients met the eligibility criteria and had household members willing to participate. Individuals who were not enrolled in KCHS were referred back to the NTLP for the completion of their medical care.

#### Delay interval

The dependent variable was a patient’s appraisal delay. It was constructed from two variables: the number of days after the appearance of the most recent symptom and the number of days from the appearance of the initial symptom to the first point of contact with the NTLP. It is an interval construction, where the number of days after the most recent symptom is always less than or equal to the number of days after the appearance of the initial symptom. Equality indicates no appraisal delay. Rather than delay only being defined as a specific number of days since the appearance of one symptom, this approach allows us to model the appraisal delay as occurring within a range, where appropriate, for patients reporting multiple symptoms occurring over a period of time. There were twenty-eight possible symptom categories from which these intervals were constructed. Numbers of days’ delay reported for each symptom were self-reported by patients upon clinical intake.

#### Geographic distance

ArcGIS® Network Analyst was used to determine the network travel time using a Kampala road network obtained from a local office [22]. OpenStreetMap was used to supplement the road network for recruited patients living outside the study catchment area [23]. Network travel time was computed from road distance and speed, as the fastest (least costly) driving network travel time in minutes from the patient home to the TB clinic. Surrogate speeds were applied where speed limits were not available and averaged actual peak time travel speeds were used to reflect traffic congestion. Pedestrian network travel time was computed similarly, using a standard travel speed of three kilometers per hour.

**Fig. 2:**
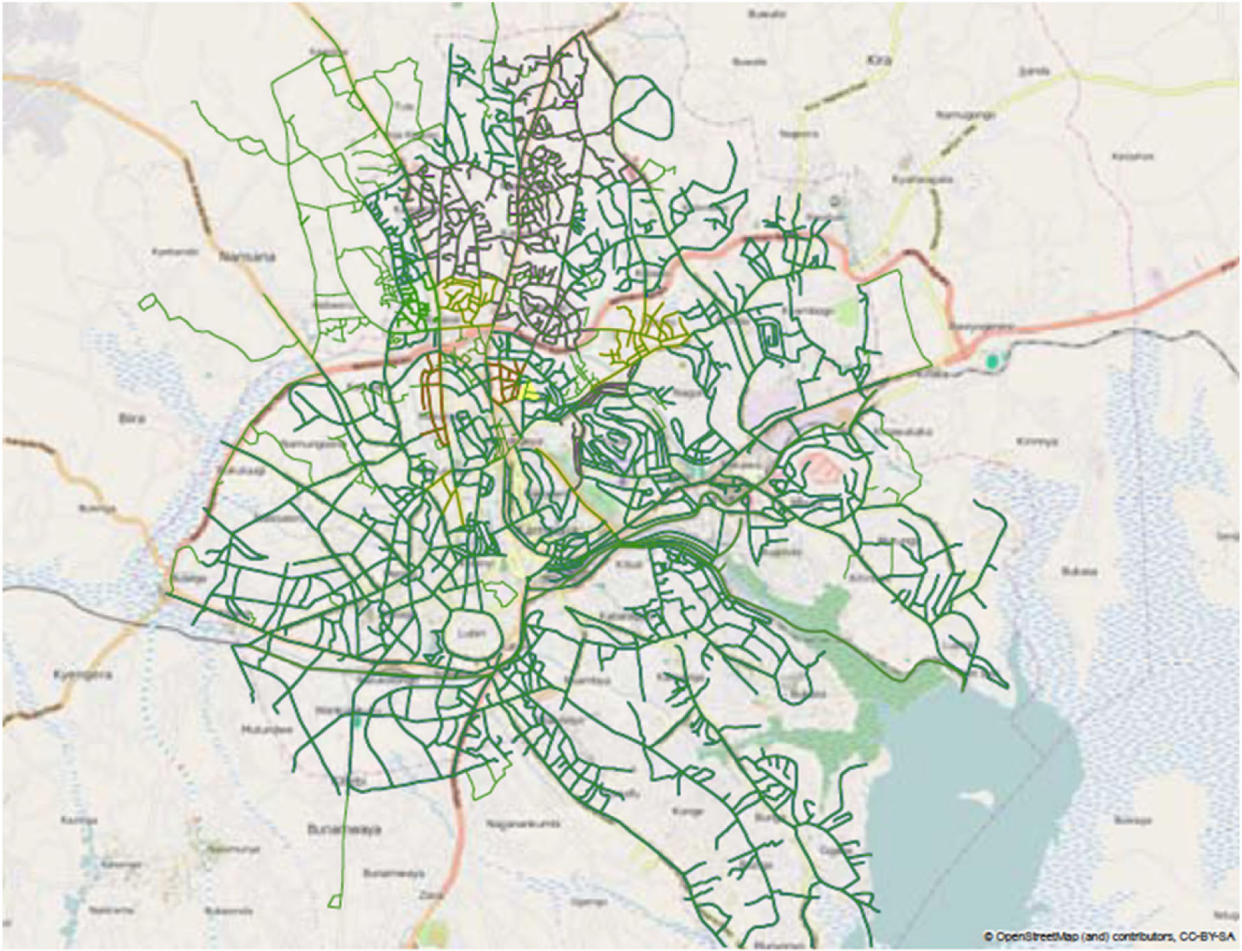
Dark green paths identify roads. Travel speeds were highlighted around areas including study households (not identified). In order of speeds, highlighted yellow and purple paths indicate higher travel speeds. The yellow square identifies the NTLP clinic. The most variable road speeds in the map are those surrounding this clinic. The Kampala, Uganda digitized base map was sourced from the Uganda Bureau of Statistics in 2009 and displayed in ArcGIS [22]. OpenStreetMap was used to supplement road travel speeds in areas not covered by the digitized maps [23]

ArcGIS® Proximity tool was used to compute the Euclidean distance as a straight-line distance from the patient home to the NTLP clinic. Detailed logistical descriptions of the ArcGIS® software and extensions can be found in this resource [24]. Figure 2 displays the Kampala road map. Yellow and purple roads indicate higher travel speeds. The yellow square identifies the NTLP clinic. The most variable speeds in the map are those surrounding this clinic.

#### Covariates

Twenty-five covariates were selected to assess the relationship of delay and geographic distance as well as to identify potential risk factors. The covariates were organized according to the Andersen utilization conceptual framework [25]:

- predisposing characteristics (age, sex, tribe, religion, marital status); enabling (patient education, social support: family size, type of residence such as Muzigo (i.e., typical housing structure for slum area) or a multi-family housing unit [26]);
- perceived needs: indicator variable describing if cough was the most recent reported symptom, total number of symptoms reported and whether the patient or any other household members were previously treated for TB;
- evaluated needs: AFB smear, chest cavities, physical examination findings (body mass index (BMI) & BCG vaccination scar), Karnofsky performance score, modified Bandim TBscore for disease severity, comorbidities: HIV status;
- and personal health practices: smoking, drinking alcohol

The Karnofsky score was segmented by a threshold score of 80, which distinguishes between patients who are able to carry on normal activity and to work, and those who are unable to work [27]. The Bandim TB score was included to assess disease severity [27]. The derivation, use and analysis of a modified version of this score are presented in the Additional file 1.

### Data analysis

Descriptive analyses comprised continuous variables expressed as median and interquartile range (IQR). Categorical variables were expressed in proportions. Chi-square and *t*-tests were used to evaluate potential differences in enabling, predisposing, evaluated and self-perceived needs and personal health behaviors as well as the delay endpoints between patients with and without GPS data. We used interval regression to assess the association between distance and delay. We distinguished between mean and variance effects on the delay outcome by estimating an interval regression model with multiplicative heteroscedasticity [28]. Estimating the variance allows us to assess how the boundaries of the delay interval change in relation to the mean. The mean delay and the log of the variance in delay were each specified as linear functions of the regressors. Estimation was by maximum likelihood (ML) with robust standard errors.

The interval regression model is specified as follows. We let *y* = *Xβ* + *ε* be the interval regression model, where *y* represents the unobserved continuous delay outcome and the *X* indicates a matrix of our covariates of interest. The model assumes *ε* ∼ N (0, *σ*^2^). For observations *j* ∈ *C*, we observe true *y*_*j*_, that is, point data for individual *j*. These uncensored delays occur either when patients report multiple symptoms with the same number of days’ duration or only one symptom. In the latter case, the most recent symptom is the initial symptom, rendering the interval to be point data. Delays that are represented by these point data suggest no appraisal delay. Observations *j* ∈ *I* are intervals. We know that the unobserved *y*_*j*_ is in the interval [*y*_*1j*_, *y*_*2j*_]. These observations include patients reporting multiple symptoms with different days’ duration associated with each one. The model assumes no right- or left-censoring.

The likelihood is proportional to the probability of observing the data, treating the parameters of the distribution as variables and the data as fixed. The goal of ML methods is to find the estimate(s) of the parameter(s) that maximizes the probability of observing the data we have. The log-likelihood of the interval regression model is specified as

$$ \ln L=-\frac{1}{2}\sum \limits_{j\in \mathrm{C}}\left\{{\left(\frac{y_j- x\beta}{\sigma}\right)}^2+\log 2\pi {\sigma}^2\right\}+\sum \limits_{j\in \mathrm{I}}\log \left\{\varPhi \left(\frac{y_{2_j}- x\beta}{\sigma}\right)-\varPhi \left(\frac{y_{1_j}- x\beta}{\sigma}\right)\right\} $$

where *Φ* is the standard cumulative normal.

Three interval regression models using the log-likelihood were analyzed: an intercept-only model, a multivariable (MV) model without multiplicative heteroscedasticity and a final multivariable model where the log of the variance was specified as linear functions of the regressors (MV + MH). The ML parameters (*β*, ln(*σ*)) for each model were compared. Additionally, we used these parameters to calculate (1) the expected delay for each individual, conditional on it being within the defined interval, and (2) the probability that the expected delay would fall in the observed interval. We posited that the final MV + MH model would maximize the mean probability of observing our data. A test of equivalence was used to assess the expected delay and probabilities of the MV + MH model [29].

To determine the set of covariates included in the final multivariable model, a series of bivariate interval regression models were fit. Significant variables (at *p* ≤ 0.10) from these models were included in the multivariable model. All distance variables were included in the model regardless of the statistical significance of their association with the delay outcome. The same variable set was used to specify the conditional variance of the delay outcome. Crude and adjusted marginal effects on delay and 95% confidence intervals (CI) were reported. An alpha of 0.05 was used for the threshold of statistical significance of primary distance predictors in the multivariable models. Probit plotting (e.g., using normal Q-Q plot) was used to assess normality of the residuals [30]. The statistical analyses were performed using Stata, the Statistics/Data Analysis statistical package, version 13 [31].

Ethical approval for the research was provided to the Tuberculosis Research Unit (TBRU) based in Case Western Reserve University and received from Institutional Review Boards at University Hospitals of Cleveland in Cleveland Ohio, USA and Uganda Council for Science and Technology in Kampala, Uganda. Participant consent was written.

## Results

### Overall description of study participants

**Fig. 3:**
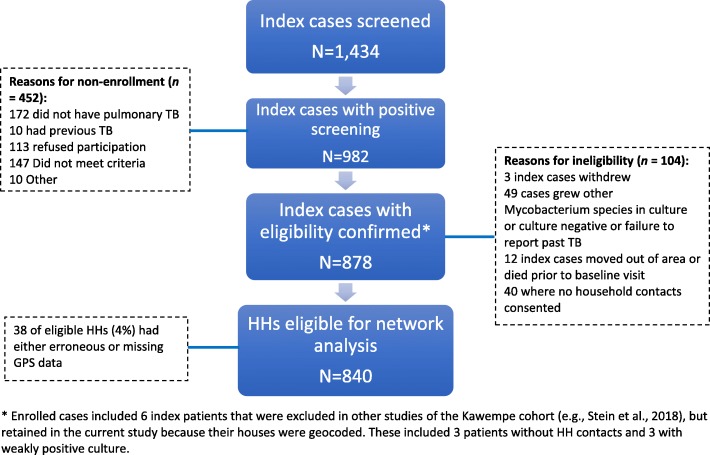
There were 984 households enrolled in the study. Of these, 878 (89%) were eligible for analysis. Of these eligible households, 840 (96%) had global positioning system waypoints available, making them eligible for inclusion in the network analysis sample

Figure 3 identifies the enrollment and eligibility flow for the Kawempe cohort study. A total of 878 newly diagnosed TB cases was enrolled during the period of April 2002 to July 2012. All but two of these eligible cases (who were missing all symptom reports) were retained in defining the interval delay outcome (see below). Of the 878 individuals, thirty-eight (4%) had either erroneous or missing GPS data. This resulted in 838 eligible TB cases in the network analysis. Among the interval patient delays, none were left- or right-censored, thereby meeting the model assumption. There were 708 patients with interval delays, producing an appraisal delay rate of 84%. Among the 838 patients with GPS data, 130 had uncensored delay with average number of 5.6 symptoms (SD = 2.3, maximum of 14) and 708 had interval delay with 7.7 symptoms (SD = 2.2, maximum of 17), a statistically significant difference (two-sample *t*-statistic = 10.5, *p* < 0.01). The only variables that were significantly different between the patient groups with (*n* = 838) and without (*n* = 38) GPS data were: marital status (*p* = 0.03), religion (*p* = 0.01), and culture result (*p* = 0.04).

The distributions of interval delay by symptom category for 876 patients are listed in Table 1. The data for cough were most complete: 875 patients (99.8%) had this symptom, with 460 patients (53%) reporting a median cough duration of exactly 90 days. There were seven other symptom categories in which 50% or more of all patients reported experiencing. All of these additional categories had a median delay of 60 days. They included loss of appetite, chest pain, fever, production of sputum, purulent sputum, nights sweats and weight loss. For each of these categories, 20% or less of all patients identifying the symptom category reported a duration of the median length of 60 days. The median minimum delay for 838 patients used in the network analysis was 30 days (mean of 36 days with standard deviation of 40.9 and range of 0 to 365 days). The median maximum delay was 90 days (mean of 122 days with standard deviation of 122.9 and range of 10 to 999 days).

**Table 1:** Symptoms and associated patient self-reported delays

Symptom	Number of all patients (*N* = 878) with symptom (%)	Median (Min, Max) number of days with symptom	Number of patients reporting symptom at min/median/max number of days		
			Min	Median	Max
Loss of appetite	546 (62)	60 (3, 365)	1	107	7
Cough	875 (99.8)	90 (0, 999)	1	460	2
Chest pain	596 (68)	60 (1, 730)	1	99	2
Dyspnea	381 (43)	40 (0, 999)	1	17	1
Fever	670 (76)	60 (1, 730)	1	101	2
Hemoptysis	87 (10)	5 (1, 66)	13	6	1
Malaise	200 (23)	30 (3, 360)	1	74	1
Produce sputum	858 (98)	60 (2, 999)	2	153	1
Purulent sputum	665 (76)	60 (2, 730)	1	122	1
Rigors	48 (5)	29 (3, 120)	1	1	4
Night sweats	544 (62)	60 (1, 730)	3	97	1
Weight loss	723 (82)	60 (2, 730)	1	142	1
Abdominal pain	26 (5)	30 (1, 365)	2	8	2
Adenopathy	9 (1)	30 (7, 140)	2	2	1
Arthralgia	72 (8)	30 (2, 365)	1	28	1
Back pain	23 (3)	30 (7, 365)	3	6	1
Bone pain	8 (1)	45 (9, 250)	1	0	1
Confusion	0 (0)	.	.	.	.
Diarrhea	32 (6)	14 (1, 112)	1	8	1
Dizziness	7 (1)	30 (1, 30)	1	5	5
Headache	34 (4)	30 (1, 150)	2	12	1
Myalgia	27 (3)	30 (3, 365)	1	6	2
Nausea	4 (0.7)	14 (4, 60)	1	2	1
Pruritis	8 (1)	33.5 (7, 360)	4	0	1
Rash	6 (1)	30 (7, 360)	2	2	1
Vision loss	0 (0)		.	.	.
Vomiting	25 (4)	20 (1, 180)	1	1	1
Other	25 (4)	42.5 (3, 720)	1	0	1

Among the predisposing factors, TB patients were mostly young adults (median age of 27 years), unmarried (54%), men (53%) who were either Roman Catholic or Anglican (61%). The predominant tribe in this setting was the Buganda tribe (56%). Among the enabling factors, patients reported a median level of 11 years of education. Overall, households consisted of a median of three members, with 2.5 members per room. Most patients lived in Muzigos (70%) with poor ventilation. Most of the patients neither smoked (81%) nor consumed alcohol (77%).

Among the patient’s perceived need factors, 18% of patients self-reported cough being the most recent symptom. This result was significantly associated with delay of more than 2 weeks (beta = 15.5, *p* = 0.001). The median number of symptoms was seven per patient. However, this variable was not significantly associated with delay (beta = − 0.68, *p* = 0.20). Only two patients reported being previously treated for TB; this variable was therefore removed from inclusion in the regression models. The need factors evaluated by the doctor identified a majority of patients with advanced disease. Most patients had a tuberculin skin test (TST) induration greater than 10 mm (85%). A majority of the patients had evident chest disease, including 63% with cavitary TB disease; 88% with moderate to far advanced TB disease extent on chest radiographs. AFB sputum smear results were positive for 93% of the patients, with 86% producing confluent growth to innumerable colonies on media. Thirty percent of the patients were HIV-positive. The median Bandim TBscore was 6, with a maximum score of 12.

### Factors associated with interval delay in bivariate models (Table 2)

All distance variables predicted increased delay in the bivariate models; however, none reached statistical significance. Each minute of network driving travel time was associated with 0.13 (standard error (SE) = 0.09) days’ delay. At median values, this represented 6.2 total days’ delay. Each kilometer of Euclidean (self-reported) distance measure was associated with 0.78 (SE = 1.03) days’ delay. At median values, this represented 2.2 total days’ delay.

**Table 2:** Bivariate associations with delay interval (in days)

Treatment-seeking characteristic	Overall median or frequency (%) (*N* = 878)	Marginal change in number of days’ delay (95% CI)	P
Geographic distance			
Pedestrian network travel time (minutes) (*N* = 840)			
Median (IQR) [Min/Max]	74.6 (56.0) [7.4/236.0]	0.05 (−0.05, 0.15)	0.35
Driving network travel time (minutes) (*N* = 840)			
Median (IQR) [Min/Max]	47.3 (25.9) [5.7/158.5]	0.13 (−0.05, 0.30)	0.16
Euclidean distance (kilometers) (*N* = 840)			
Median (IQR) [Min/Max]	2.78 (2.36) [0.24/10.78]	0.78 (−1.24, 2.80)	0.45
Self-reported distance (*N* = 865) (kilometers)			
Median (IQR) [Min/Max]	5 (5) [0/20]	0.87 (−0.18, 1.92)	0.10
Predisposing factors			
Sex			
Male	464 (53%)	−3.32 (−9.65, 3.0)	0.30
Female	414 (47%)	Ref	
Age (in years)			
Median (IQR) [Min/Max]	27 (12) [18/80]	0.60 (0.03, 1.12)	0.04
Married			
Yes	406 (46%)	3.04 (−3.34, 9.43)	0.35
No	472 (54%)	Ref	
Tribe			
Buganda	496 (56%)	−1.86 (−8.21, 4.49)	0.57
Other	382 (44%)	Ref	
Religion			
Roman Catholic (RC)	273 (31%)	−3.26 (−13.2, 6.63)	0.52
Anglican	263 (30%)	7.10 (−4.2, 18.5)	0.22
Muslim	203 (23%)	−0.82 (−11.1, 9.5)	0.88
Other	138 (16%)	Ref	
Enabling Factors			
Education (years) (*N* = 877)			
Median (IQR) [Min/Max]	11 (7) [1/17]	−0.75 (−1.51, 0.02)	0.06
Household size (*N* = 874)			
Median (IQR) [Min/Max]	3 (3) [1/30]	−0.05 (− 1.76, 1.66)	0.96
Person/room			
Median (IQR) [Min/Max]	2.5 (2.43) [0/14]	−0.18 (−2.05, 1.69)	0.85
Windows/room			
Median (IQR) [Min/Max]	0.8 (1) [0/2.5]	−0.35 (−6.47, 5.78)	0.91
Type of residence			
Muzigo	613 (70%)	−1.43 (−8.59, 5.71)	0.70
Other	265 (30%)	Ref	
Need factors			
Perceived by patient			
Cough most recent symptom			
Yes	158 (18%)	15.49 (6, 25)	0.001
No	720 (82%)	Ref	
Total number of symptoms (*N* = 874)			
Median (IQR) [Min/Max]	7 (3) [0/23]	−0.68 (−1.71, 0.35)	0.20
Need factors			
Evaluated by doctor			
Modified Bandim TB score			
Median (IQR) [Min/Max]	6 (2) [0/12]	0.31 (−1.40, 2.01)	0.73
TST Cut-off 10 mm			
Positive	644 (85%)	−4.03 (−16.3, 8.27)	0.52
Negative	111 (15%)	Ref	
BCG Vaccination			
Yes	492 (65%)	2.52 (−2.51, 7.56)	0.33
No	264 (35%)	Ref	
BMI (*N* = 876)			
Median (IQR) [Min/Max]	19 (3.2) [12.4/35.5]	−0.83 (−1.83, 0.16)	0.10
HIV Status			
Positive	265 (30%)	−5.65 (−12.2, 0.86)	0.09
Negative	612 (70%)	Ref	
Index Karnofsky			
≥ 80	798 (91%)	0.11 (−0.38, 0.60)	0.66
< 80	80 (9%)	Ref	
Cavitary disease			
Yes	539 (63%)	9.50 (3.30, 15.70)	0.003
No	321 (37%)	Ref	
Cavitary disease extent^a^			
Far advanced	455 (53%)	10.47 (1.04, 19.90)	0.03
Mod advanced	304 (35%)	0.45 (−9.43, 10.33)	0.93
Normal or Minimal	101 (14%)	Ref	
AFB Grade smear			
3+	713 (81%)	11.12 (2.3, 19.94)	0.01
2+	106 (12%)	12.29 (−2.4, 27))	0.10
0–1+	59 (7%)	Ref	
Culture result			
50+ colonies	588 (68%)	9.03 (−0.3, 18.35)	0.06
30–49 colonies	155 (18%)	9.17 (−2.5, 20.84)	0.12
0–29 colonies	128 (15%)	Ref	
Personal health behaviors			
Smoking			
Current or former smoker	164 (19%)	5.29 (−1.3, 11.85)	0.12
Never smoke	713 (81%)	Ref	
Alcohol intake			
Yes	204 (23%)	−4.06 (−10.6, 2.44)	0.22
No	673 (77%)	Ref	

*Ref* Reference category, *TST* tuberculin skin test

^a^Reference group comprised normal (*N* = 28) and minimal (*N* = 73) cavitary disease

Among the predisposing factors, only older age significantly predicted increased delay: being 1 year older predicted 0.60 days’ delay (95% confidence interval (CI): 0.03, 1.12). Of the enabling factors, more years of education marginally predicted reduced days’ delay: 1 year of additional education was associated with reduction in delay by 0.75 days (95% CI: -1.5, 0.02). Among the need factors from the patient’s perspective, cough being the most recent symptom (i.e., cough duration was equivalent to the minimum delay value of the appraisal interval) was associated with more than 2 weeks’ delay (15.5 days) (95% CI: 6, 25). This covariate was the most significant predictor of delay (*p* = 0.001) among the full set of covariates considered. Other need factors evaluated by a doctor also predicted increased number of days’ delay. Patients with cavitary disease experienced almost 10 days’ delay (*p* = 0.003), especially patients with far advanced disease (*p* = 0.03) and high AFB Grade smear (*p* = 0.01). No personal behaviors were significantly associated with delay. A higher Bandim TBscore was associated with increased delay, although the result was not statistically significant (beta = 0.31, *p* = 0.73). We further consider analysis of the TBscore, including its significant association with the delay variance, in the Additional file 1.

### Factors associated with interval delay in multivariable model (Table 3)

The final multivariable model included a sample of 798 observations, consisting of 123 uncensored delays and 675 interval observations, representing 89 and 91% of the available patient data on delay. Nine covariates deemed significant (*p* ≤ 0.10) from the bivariate results were included in the MV + MH model. These included patient age, years of education, cough being the most recent symptom, BMI, HIV status, cavitary disease, cavitary disease extent, AFB grade smear and culture result. For interpretation of the model intercept, age and BMI were mean-centered. In this adjusted model, there was a more pronounced and greater effect of distance on delay. Driving network travel time significantly predicted increased delay (*p* = 0.02): each minute of driving time was associated with 0.25 days’ delay (95% CI: [0.07, 0.44]). At median values, this represented 11.8 days’ delay. Thus, adjusting for other patient and clinical factors, the median driving time added 12 (95% CI: [3, 21]) days to the average patient delay of 40 days (95% CI: [25, 56]), an increase of 30%. However, increasing Euclidean distance was associated with reduced variability in the delay interval (beta = − 0.32, *p *= 0.02). Adjusting for the same factors, at the median Euclidean distance of 2.8 km, the variance in the delay was reduced by more than 25% (beta × median distance / constant). These results demonstrate that while driving time influenced changes in the mean delay, Euclidean distance was associated with precision of the delay interval length.

**Table 3:** Multivariable associations with appraisal delay interval (in days) using multiplicative heteroscedasticity

Treatment-seeking characteristic	Change in # of days’ delay (95% CI)	P	Change in variance of days’ delay (95% CI)	P
Geographic distance				
Pedestrian network travel time (min)	−0.05 (−0.41, 0.32)	0.79	0.009 (− 0.002, 0.02)	0.10
Driving network travel time (min)	0.25 (0.05, 0.47)	0.02	0.003 (−0.002, 0.01)	0.25
Euclidean distance (km)	−2.54 (−10.3, 5.24)	0.52	−0.32 (− 0.56, − 0.06)	0.02
Self-reported distance (km)	0.95 (− 0.13, 2.02)	0.09	0.01 (− 0.01, 0.04)	0.30
Predisposing factors				
Age (in years)	0.64 (0.31, 0.98)	<.001	0.02 (0.01, 0.03)	<.001
Enabling Factors				
Education (years)	−0.50 (−1.16, 0.16)	0.14	−0.02 (− 0.03, 0.002)	0.08
Need factors				
Perceived by patient				
Cough most recent symptom				
Yes	13.35 (4.67, 22.02)	0.003	0.50 (0.29, 0.70)	< 0.001
No	Ref		Ref	
Need factors				
Evaluated by doctor				
BMI	−0.54 (−1.36, 0.28)	0.20	−0.03 (− 0.05, −.004)	0.02
HIV Status				
Positive	−0.70 (−7.2, 5.83)	0.83	0.05 (−0.16, 0.26)	0.66
Negative	Ref		Ref	
Cavitary disease				
Yes	8.99 (2.20, 15.79)	0.009	0.31 (0.09, 0.53)	0.006
No	Ref		Ref	
Cavitary disease extent^a^				
Far advanced	3.29 (−8.90, 15.48)	0.60	−0.23 (−0.55, 0.11)	0.18
Moderately advanced	−5.13 (−16.03, 5.79)	0.36	−0.18 (− 0.49, 0.12)	0.23
Normal or Minimal	Ref		Ref	
AFB Grade smear				
3+ = 3	−1.0 (−12.43,10.45)	0.86	−0.05 (− 0.48, 0.38)	0.83
2+ = 2	6.21 (−7.6, 20.03)	0.38	0.49 (0.002, 0.98)	0.05
0–1+ = 1	Ref		Ref	
Culture result				
50+ colonies	12.49 (5.01, 19.97)	0.001	0.41(0.16, 0.66)	0.002
30–49 colonies	13.63 (4.17, 23.10)	0.005	0.32 (0.003, 0.62)	0.05
0–29 colonies	Ref		Ref	
Constant	39.5 (25.3, 55.7)	< 0.01	3.17 (2.67, 3.66)	< 0.001

^a^Reference group comprised normal (*N* = 28) and minimal (*N* = 73) cavitary disease

*Ref* Reference category

Overall, the log-likelihood for the fully specified MV + MH model (− 1318) suggested a better fit, compared to − 1426 for the MV model and − 1608 for the intercept-only model. Sensitivity checks revealed the robustness of these main effects. The statistical significance of the driving time predictor was attenuated (*p* = 0.07) and the marginal effect was reduced by more than 20% when not also modeling the delay variance. This supports the importance of accounting for the variability associated with delay in urban areas with locations of particularly congested traffic zones. Second, in validating assumptions of the MV + MH model, we observed non-normality of the residuals using a normal Q-Q plot. To inspect the impact of this violation, we re-analyzed the model including only observations whose residuals were within the interquartile range of the distribution (*N* = 399). This check revealed a 50% increase in the effect estimate for driving time (beta = 0.38, *p* < 0.001). Furthermore, self-reported distance in kilometers was also now statistically significant (beta = 1.1, *p* = 0.003). These results revealed that driving time as a distance metric was more robust to model misspecification than other measures.

Among the other covariates included in the multivariable model, several significantly (*p* ≤ 0.05) and positively tracked both mean delay and the variability in the interval: increasing patient age, cough being the most recent symptom reported, presence of cavitary disease, and higher culture result. Using the median value, continuous age was associated with 17.3 days’ delay, cough being the most recent symptom indicated 13.4 days’ delay, and having advanced disease contributed between nine (for cavitary disease) to 12.5 (for culture result of 50+ colonies) days’ delay. Collectively assessed, older and sicker patients accumulated the greatest appraisal delay. Controlling for these patient and disease characteristics, driving time distance significantly modified the mean delay outcome.

**Fig. 4:**
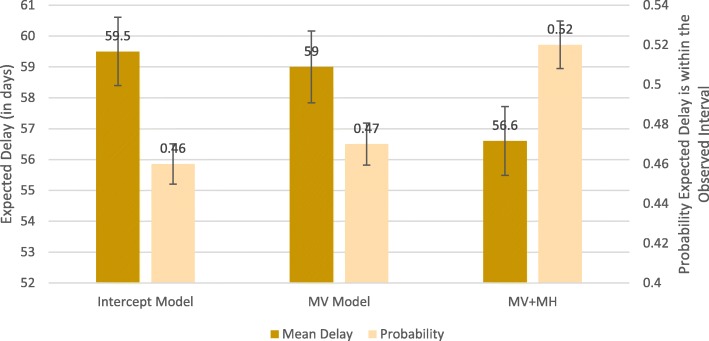
The expected delay (in days) was calculated for each patient under each model scenario. The per-patient probability that this expected value was contained in the observed delay interval was derived. The means of both outcomes are shown in the figure. Bars represent one standard error from the mean. Abbreviations: MV, multivariable model; MV + MH, multivariable model with multiplicative heteroscedasticity

Figure 4 shows the post-estimation results of the interval regression models using ML for all 675 interval delay observations. The expected delay (in days) was calculated conditional on the value being within the interval identified for each individual. The mean probability that this expected value was contained in the interval observed in the data is also shown. For the intercept model, the MV model and the MV + MH model, the respective estimates were as follows: 59.5 days with probability 0.46, 59 days with probability 0.47 and 56.6 days with probability 0.52.

A test of equivalence was performed on the mean estimates from the MV and MV + MH models. To define the equivalence margins, we used the standard deviations of the estimate differences between the intercept and MV models (0.05 for probability and 4 days in delay). Results revealed equivalence in the delay outcome, but not the probability. The ML estimates that achieved the 0.52 probability are contained in Table 3. Modeling the variance of the delay outcome therefore significantly increased the probability that we observed our data.

## Discussion

Our findings suggest that adjusting for TB disease severity, patients with longer driving times to access TB treatment may be more vulnerable to delay and its concomitant morbidity. However, this association was not present when omitting these disease status characteristics in the bivariate model. That is, distance in driving time was not statistically associated with the appraisal delay outcome among our cohort of patients, most of whom had advanced disease. This result conforms to Stock’s [19] assessment on delay and distance, suggesting patients with less serious disease are prone to delay as a result of the barriers associated with driving time.

Our patients present a particular public health challenge as longer delay results in continued MTB exposure and transmission. Given the available GIS software, TB control programs can identify populations experiencing greater travel times (which may not reflect distance traveled) and provide the appropriate interventions in order to reduce the travel burden. Our novel application of interval regression with multiplicative heteroscedasticity provides an estimate of the effect of the main predictors on mean delay, while also taking into account the increased variability associated with shorter kilometer distance from the clinic.

A number of previous studies [9, 32–34] have identified geographic distance as an important factor for delay. However, most of the studies perform a cursory assessment of distance (e.g., urban versus rural or only self-reported travel time or distance). This study increases the rigor and sophistication by using both network travel time and Euclidean distance to characterize a more complete picture of the impact of distance on delay. The Euclidean distance measure alone, though computationally simple, may be limited in settings of high traffic congestion [35]. Indeed, in this study where traffic congestion is common in areas close to the clinic, we found that shorter Euclidean distance predicted increased variability of delay in the multivariable analyses (likely dependent on the designated road speeds intended to accommodate such congestion). In contrast, increasing network travel driving time was associated with increased patient delay. These findings were consistent with previous research that closely examined geographic distance and delay [36]. Among the predisposing factors examined in this study, increasing patient age (but not sex) were significant risk factors for delay, mostly aligning with existing literature [33, 37, 38]. Although more years of education were protective against delay, our results were not significant, matching others who found no relationship between education and delay [34, 39]. However, almost all patients reported cough and the recentness of this symptom was positively and significantly associated with delay. Notably, this is a considerably higher proportion than that reported in other sub-Saharan nations [40, 41], but likely reflects our cohort’s more advanced disease.

### Study limitations

The study represented individuals selected for research purposes according to the inclusion and exclusion criteria which would limit the generalizability to a population with similar characteristics. Among these criteria was the inclusion of mostly newly diagnosed TB patients. Patients with recurrent episodes of TB may experience a different set of risk factors based on their previous knowledge of TB disease and encounters with the health system. Patients with recurrent TB constituted less than 1 % of our study sample. Sensitivity analyses revealed that our results did not change by excluding these patients; however, future studies should further assess what impact recurrent TB has on our understanding of distance on treatment delay. Furthermore, the study enrolled patients within Kawempe and contiguous counties in a 20 km radius; this may also limit generalizability to similarly urban and congested areas.

We used the earliest and most recent number of days since the start of a list of symptoms to calculate the appraisal delay interval. Ostensibly recall bias may have played a role. This bias is further complicated by similarly presenting infectious diseases endemic in this region. To minimize the bias, patients were interviewed by trained and experienced medical doctors who correlated the presenting signs and symptoms with the patient’s disease progression. Furthermore, the derived appraisal delay interval may be biased upwards, as some patients may have sought care from other providers before their arrival at the NTLP clinic. Unfortunately, we do not have data on whether or when subjects visited other health care providers in relation to their symptom reports. However, because very few of our index cases were previously treated for TB, it is possible that any prior treatment-seeking behaviors may not have been a result of the patient’s awareness of their TB status.

## Conclusion

Our study finds that geographic distance was associated with delay. Of the four geographic distance measures, network travel driving time was a better and more robust predictor of mean delay in this setting. We find that increasing network travel driving time increases the number of days’ delay. Other important contributors to delay include patient age and disease progression. We conclude that, in addition to the use of traditional risk factors, TB control programs should consider network travel time in identifying vulnerable populations, with the caveat that increasing variability in congested areas may make it more difficult to discern the influence of distance on patient appraisal delay.

## Additional file

Additional file 1: Adaptation and analysis of the TBscore in the Kawempe Community Health Cohort Study. (DOCX 30 kb)

## Abbreviations

AFB: Acid-fast bacilli
BCG: Bacillus Calmette–Guérin
BMI: Body mass index
FMI: Fat mass index
GPS: Global positioning system
HIV/AIDS: Human immunodeficiency virus / acquired immunodeficiency syndrome
IQR: Interquartile range
KCHS: Kawempe Community Health Study
LMI: Lean mass index
ML: Maximum likelihood
MUAC: Mid-upper arm circumference
NTLP: National Tuberculosis and Leprosy Program
TB: Tuberculosis
TBRU: Tuberculosis Research Unit
TST: Tuberculin skin test
