# Supplementary material for: Impact of geographic distance on appraisal delay for active TB treatment seeking in Uganda: a network analysis of the Kawempe Community Health Cohort Study
Source: BMC Public Health. 2018 Jun 26;18:798. doi: 10.1186/s12889-018-5648-6 (PMC6019214; doi:10.1186/s12889-018-5648-6)
Supplement: Supplementary file 1 — Adaptation and analysis of the TBscore in the Kawempe Community Health Cohort Study. (DOCX 30 kb) [file 12889_2018_5648_MOESM1_ESM.docx]

**Impact of geographic distance on appraisal delay for active TB treatment seeking in Uganda: a network analysis of the Kawempe Community Health Cohort Study**

*Supplementary Material*

*Deriving a modified TBScore*

The Bandim TB score was included as a covariate to control for a patient’s disease severity [1]. A high score serves as a proxy for disease severity, which has been shown to be associated with delay [2]. The original and follow-up derivations of the score use as a component the mid-upper arm circumference (MUAC) [1, 3]. In the score, a patient gets one point if he or she has a MUAC < 220 mm, and if he/she has a score < 200 mm, he/she gets an extra point [1]. However, this component was not available in the current dataset. A proxy for this missing component measure is described next.

Al-Gindan et al. [4] showed that MUAC correlated well with lean body mass as determined by whole-body magnetic resonance imaging, especially in men. In a sample of non-pregnant women, low MUAC was associated with altered lean tissues (whereas fat tissue did not differ) [5]. Arm anthropometry correlates strongly with fat mass (but less so with lean mass) in children [6]. However, because fat mass is also a significant predictor of TB severity in sub-Saharan Africa [7], we retained both lean and fat mass indices to produce a modified TBscore calculation.

The lean and fat mass indices for the Kawempe cohort study were calculated in Mupere et al. [8]. To align with previous methodology [1-3, 9], we replaced MUAC with lean mass index (LMI) and fat mass index (FMI). If patients had a low LMI (<16.7 (kg/m^2^) for men and <14.6 (kg/m^2^) for women), then they received one point; if they also had low FMI (low FMI (<1.8 (kg/m^2^) for men and <3.9 (kg/m^2^) for women) then they received another point. Note that only having low FMI (but not low LMI) earned zero points. Even with the scoring change, the maximum possible score was still thirteen.

This revised algorithm was used on the basis that in this Ugandan sample, LMI has been associated with both mortality and recovery from TB. Mupere et al. [10] showed that Ugandan women, but not men, with low baseline LMI had a five-fold greater hazard of death. However, Mupere et al. [8] showed that wasted male tuberculosis patients regained LMI at a greater rate than FMI during initial tuberculosis therapy.

Among the 878 index cases included as part of this study, 171 patients were missing at least one mass measure. This meant that their TBscore was incomplete. To assess whether this biased the calculation, we used a t-test to compare the scores. Patients with these missing elements had a mean TBscore of 5.8 (SD = 1.71), whereas patients with complete data had a mean TBscore of 6.0 (SD = 2.21). A two-sample t-test with unequal variances suggested no significant difference between the scores (p > 0.05).

We also derived a modified version of the revised TBscoreII [3], employing the same proxy as described above. However, for the modified score, several components are excluded:  night sweats, positive lung auscultation findings, pulse, temperature, and hemoptysis. Thus, the maximum possible score was eight. For the same sample size, patients with a missing fat and/or lean mass measure had a mean TBscoreII of 2.73 (SD = 1.20), whereas patients with no missing data had a mean TBscoreII of 3.10 (SD = 1.65). A two-sample t-test with unequal variances suggested a significant difference between the scores (p < 0.01). Therefore, we retain use of the original score for the main analysis and the supplemental analysis reported here.

*Applying the TBscore*

Before using the TBscore in a regression analysis, we further examined the minimum and maximum delay outcomes in relation to a binary TBscore measure, based on a median cut point (TBscore of 5 or lower versus 6 or greater). An equality-of-medians test for both delay measures (dropping delay values equal to the median from the analysis) revealed the number of patients whose minimum (but not maximum) delay outcome was greater than the median to be higher for the group whose TBscore value was below the cutoff (continuity-corrected p = 0.04). This result suggested that the Bandim TBscore exerts influence over the variability in the appraisal delay, such that patients with less severe disease had a more precise approximation of the delay interval.

Recall from the methodology described in the manuscript that the same variable set predicting the number of days’ delay was also used to specify the conditional variance of the delay outcome. The TBscore was not a statistically significant covariate in the bivariate interval regression model (p = 0.73); hence, it was not included in the multivariable model or the specification of the delay variance. However, for this exercise, we included in the final MV+MH model of the manuscript the TBscore as a covariate in predicting both days’ delay as well as the delay variance. Our goal was to assess whether its inclusion modified any of the main distance results.

Table S1 includes the results of the MV+MH model with adjustment for the TBscore. The log-likelihood for this model was -1318. A likelihood ratio test indicated the model with TBscore predicting the days’ delay and variance of this outcome produced a better model fit: the chi-square statistic with two degrees of freedom was 7.36 (p = 0.03). Further inspection of the parameter estimates revealed that each point increase in the TBscore increased the variance of delay by 0.05 (p = 0.04). This is not altogether unexpected. People who are sick longer or with more serious disease probably have more symptoms and a fair amount of difficulty in remembering when symptoms started.

Also noteworthy is the estimate for self-reported distance now significantly predicts patient delay; each kilometer in distance increases delay by just over one day (p = 0.04). These findings do not alter the significant associations presented in the main manuscript i.e., increased driving time distance positively predicts delay and increased Euclidean distance reduces variability of delay. Overall, controlling for the TB score generates parallel results for self-reported distance and driving distance in the mean model, suggesting that greater distance (as assessed with multiple measures) significantly increases delay in treatment for TB.

The major limitation of this TBscore adaptation is an inability to collect additional data on both MUAC and LMI/FMI to see if and how the adjustments we have proposed equally predict the TBscore outcome. This means addressing both proxies in turn. First, determining whether low LMI (<16.7 (kg/m^2^) for men and <14.6 (kg/m^2^) for women) is correlated with MUAC of less than 220 mm. Second, identifying whether both low LMI and low FMI (<1.8 (kg/m^2^) for men and <3.9 (kg/m^2^) for women) are correlated with MUAC of less than 200 mm.

Table S1. Inclusion of TBscore in the MV+MH model to assess potential changes in the main distance measures. Note that not all covariates in the model are reported in the table.

| Treatment-seeking characteristic | Change in # of days’ delay (95% CI) | P | Change in variance of days’ delay  (95% CI) | P |
| --- | --- | --- | --- | --- |
| Geographic distance:  Pedestrian network travel time (min)  Driving network travel time (min)  Euclidean distance (km)  Self-reported distance (km)  Bandim TBscore | -0.02 (-0.38, 0.35)  0.25 (0.05, 0.45)  -3.45 (-11.23, 4.33)  1.10 (0.04, 2.15)  0.46 (-1.17, 2.10) | 0.93  0.01  0.38  0.04  0.58 | 0.009 (-0.002, 0.02)  0.004 (-0.002, 0.01)  -0.33 (-0.59, -0.07)  0.02 (-0.01, 0.05)  0.05 (0.003, 0.10) | 0.10  0.25  0.01  0.18  0.04 |

Supplement References

1. Wejse C, Gustafson P, Nielsen J, Gomes VF, Aaby P, Andersen PL, Sodemann M. TBscore: Signs and symptoms from tuberculosis patients in a low-resource setting have predictive value and may be used to assess clinical course. Scandinavian journal of infectious diseases. 2008 Jan 1;40(2):111-20.
2. Virenfeldt J, Rudolf F, Camara C, Furtado A, Gomes V, Aaby P, Petersen E, Wejse C. Treatment delay affects clinical severity of tuberculosis: a longitudinal cohort study. BMJ open. 2014 Jun 1;4(6):e004818.
3. Rudolf F, Lemvik G, Abate E, Verkuilen J, Schön T, Gomes VF, Eugen-Olsen J, Østergaard L, Wejse C. TBscore II: refining and validating a simple clinical score for treatment monitoring of patients with pulmonary tuberculosis. Scandinavian journal of infectious diseases. 2013 Nov 1;45(11):825-36.
4. Al-Gindan YY, Hankey C, Govan L, Gallagher D, Heymsfield SB, Lean ME. Derivation and validation of simple equations to predict total muscle mass from simple anthropometric and demographic data. The American Journal of Clinical Nutrition. 2014 Oct 1;100(4):1041-51.
5. Gartner A, Maire B, Kameli Y, Traissac P, Delpeuch F. Body composition unaltered for African women classified as ‘normal but vulnerable’ by body mass index and mid-upper-arm-circumference criteria. European Journal of Clinical Nutrition. 2001 May 1;55(5):393.
6. Chomtho S, Fewtrell MS, Jaffe A, Williams JE, Wells JC. Evaluation of arm anthropometry for assessing pediatric body composition: evidence from healthy and sick children. Pediatric Research. 2006 Jun 1;59(6):860-5.
7. Van Lettow M, Kumwenda JJ, Harries AD, Whalen CC, Taha TE, Kumwenda N, Kang'ombe C, Semba RD. Malnutrition and the severity of lung disease in adults with pulmonary tuberculosis in Malawi. The International Journal of Tuberculosis and Lung Disease. 2004 Feb 1;8(2):211-7.
8. Mupere E, Malone L, Zalwango S, Okwera A, Nsereko M, Tisch DJ, Parraga IM, Stein CM, Mugerwa R, Boom WH, Mayanja HK. Wasting among Uganda men with pulmonary tuberculosis is associated with linear regain in lean tissue mass during and after treatment in contrast to women with wasting who regain fat tissue mass: prospective cohort study. BMC Infectious Diseases. 2014 Jan 13;14(1):24.
9. Rudolf F, Joaquim LC, Vieira C, Bjerregaard-Andersen M, Andersen A, Erlandsen M, Sodemann M, Andersen PL, Wejse C. The Bandim tuberculosis score: reliability and comparison with the Karnofsky performance score. Scandinavian Journal of Infectious Diseases. 2013 Apr 1;45(4):256-64.
10. Mupere E, Malone L, Zalwango S, Chiunda A, Okwera A, Parraga I, Stein CM, Tisch DJ, Mugerwa R, Boom WH, Mayanja H. Lean tissue mass wasting is associated with increased risk of mortality among women with pulmonary tuberculosis in urban Uganda. Annals of epidemiology. 2012 Jul 31;22(7):466-73.
